# Society Meetings

**Published:** 1918-02

**Authors:** 


### SOCIETY MEETINGS

**Erie County Medical Society.** Annual meeting, Buffalo Medical College, Dec. 17. President, Dr. Irving W. Potter, called the meeting to order, and the Secretary, Dr. Franklin C. Gram, read the minutes of the previous meeting and the Council meetings, all of which were approved.

Dr. William F. Jacobs, chairman of the Committee on Membership, presented the applications of the following candidates, all of whom were separately voted upon and elected to membership:

Dr. Earl W. Thoma, Dr. Francis M. Kujawa, resident internes Buffalo General Hospital; Dr. Sergeant Price Martin, 749 Forest Avenue; Dr. Joseph Francis Shanahan, 2157 Main Street; Dr. Harold A. Patterson, 116 Goodell Street; Dr. Irving Franklin Gram, 849 Humboldt Parkway; Dr. Nathaniel Barone, resident interne, Buffalo Homoeopathic Hospital; Dr. Francis J. Butlak, 1071 Sycamore Street; Dr. Vincent S. Mancuso, 476 Front Avenue.

Reinstatement of Dr. Harry B. Pinkerton, 597 Elmwood

Avenue, Buffalo. Transfer of Dr. W. E. McChesney from Monroe County.

Treasurer Lytle presented his annual report, which showed that 692 members were in good standing at the time of making his report, and that this number would possibly be increased by a large number of members who were in arrears at the time of the report, and who will undoubtedly pay before the close of the year.

The report of the tellers, Drs. Mulford, Lytle and Reimann showed that the following were elected: Pres., Dr. George F. Cott; 1st Vice-Pres., Dr. James E. King; 2d Vice-Pres., Dr. Earl B. Lothrop; Sec., Dr. Franklin C. Gram; Treas., Dr. Albert T. Lytle; Censors, Drs. John D. Bonnar, Francis E. Fronczak, Arthur G. Bennett, Archibald D. Carpenter. Frank A. Valente. Chairman Committee on Legislation, Dr. Harvey B. Gaylord; Chairman Committee on Public Health, Dr. Nelson G. Russell; Chairman Committee on Membership, Dr. William F. Jacobs; Chairman Committee on Economics, Dr. John V. Woodruff. Delegates to the State Society: Drs. Arthur G. Bennett, George F. Cott, Julius Richter, Franklin W. Barrows. Delegates holding over for 1918: Drs. Grover W. Wende, Harry R. Trick, Arthur W. Burd, Irving W. Potter, Charles G. Stockton.

Dr. Woodruff submitted his annual report as Chairman of the Committee on Economics. This report contained the following recommendations:

1. That the by-laws of the New York State Medical Society be changed or amended so as to give the county societies the right to home rule in order that they may regulate their own affairs in accordance with their special needs and requirements.

2. That our Committee on Legislation be instructed to use its utmost endeavors to have the Compensation Law so amended that the individual employee may have the inalienable right vouchsafed him by the Constitution to choose his own medical or surgical attendant without let or hindrance from the employer.

On motion of Dr. Rochester the report was received and the recommendations contained therein adopted.

Dr. Thomas H. McKee, Chairman of the committee appointed to visit the various Medical Societies within the county to discuss with them the subject of increasing medical fees to meet present economic conditions, reported progress and asked for the continuation of his committee. The report was accepted and the committee continued.

Dr. John D. Bonnar, Chairman of the Board of Censors, submitted his annual report giving a detailed account of the work performed by this board during the year. The report was accepted and the financial statement contained therein was referred to the council for audit.

Dr. Clayton W. Greene, secretary of the Erie County Milk Commission, submitted the annual report of the commission.

Dr. Franklin W. Barrows, Chairman of the special committee on the Akron Home for Feeble Minded, made a verbal report for this committee.

The resolutions adopted by the Auburn Academy of Medicine, relative to the repeal of the State Narcotic Law, were read by the secretary, and on motion of Dr. Woodruff were endorsed by the society.

On motion of Dr. Gram the president was requested to appoint a committee of three to draw up a suitable statement, which would express the views of the society, relative to the State Narcotic Law and its repeal.

Treasurer Lytle stated that at a previous meeting of the society the question of remitting dues of members who had gone to the front had been referred to the council with power. This would affect approximately 135 members, who were thus far known to be in the service of the U. S., and this number would undoubtedly be greater by next year. This question had been seriously considered by the council, and a recommendation had been adopted requesting the State Society to suspend the state dues of all members who had gone to the front, and to continue such suspension during the continuance of the war. At a meeting of the State Council this question had been considered, and it was determined that under the by-laws the State Council had no right to suspend this by-law, and that it would require action by the House of Delegates at the next meeting of the State Society. However, the council had taken action which would continue in good standing those members who are in arrears with their dues.

Dr. Irving W. Potter then delivered a brief address as the retiring president, in which he thanked the members, officers and various committees for their splendid cooperation during the year, and then introduced the newly elected president, Dr. George F. Cott, who made a brief speech thanking the society for the honor, and outlined his policy for the coming year.—Franklin C. Gram, Secretary.

---

**The Gross Medical Club** held its bi-monthly meeting at the Hotel Statler, Buffalo, Dec. 21, Dr. Jas. E. King, President, acting as host. The paper ''Inflammation in the female pelvis due to infection from without,'' was read by Dr. J. Henry Dowd, and will be published in a later issue. The paper was discussed by all members present, the consensus of opinion being, remove all sources of infection in the male before allowing them to marry or resume sexual relations, and infections in the female would be reduced to the minimum.

Dr. Irving Potter reported two most interesting cases of placenta previa. In one he was called in consultation early and performed Caesarian section at once, saving both mother and child. In the other he was not called for 24 hours after haemorrhage had started; operation was done at once but both mother and child died.

Dinner was served in the main dining room, there being 37 present.

---

**American Congress on Internal Medicine,** second scientific session was held in Pittsburgh, Pa., on December 27 and 28, 1917. The following papers were read: Dr. Charles D. Aaron, Detroit, Roentgenology and the Internist; Major George C. Johnston, Pittsburgh, The Roentgen Rays in the Diagnosis of Diseases of the Thoracic Cavity; Dr. William A. Evans, Detroit, Roentgen Ray Diagnosis of Abdominal Diseases; Dr. Russell H. Boggs, Pittsburgh, The Value of the Roentgen Ray and Radium in the Treatment of Internal Disease; Dr. William H. Park, New York, The Communicable Diseases in Modern Warfare and their Prevention; Dr. Frank Smithies, Chicago, The Realationship of Infection in the Production of so-called Pernicious Anemia and its Significance with respect to Treatment; Dr. Edward E. Cornwall, Brooklyn, Some Problems of Cardio-Vascular Disease.

